# Targeting Filamenting temperature-sensitive mutant Z (FtsZ) with bioactive phytoconstituents: An emerging strategy for antibacterial therapy

**DOI:** 10.1371/journal.pone.0290852

**Published:** 2023-08-30

**Authors:** Bader Saud Alotaibi

**Affiliations:** Department of Laboratories Sciences, College of Applied Medical Sciences, Shaqra University, Alquwayiyah, Saudi Arabia; University of Sadat City, EGYPT

## Abstract

The rise and widespread occurrence of bacterial resistance has created an evident need for novel antibacterial drugs. Filamenting temperature-sensitive mutant Z (FtsZ) is a crucial bacterial protein that forms a ring-like structure known as the Z-ring, playing a significant role in cell division. Targeting FtsZ is an effective approach for developing antibiotics that disrupt bacterial cell division and halt growth. This study aimed to use a virtual screening approach to search for bioactive phytoconstituents with the potential to inhibit FtsZ. The screening process proceeded with the filtering compounds from the IMPPAT library of phytochemicals based on their physicochemical properties using the Lipinski rule of five. This was followed by molecular docking, Pan-assay interference compounds (PAINS) filter, absorption, distribution, metabolism, excretion, and toxicity (ADMET), prediction of activity spectra for biologically active substances (PASS), and molecular dynamics (MD) simulations. These filters ensured that any adverse effects that could impede the identification of potential inhibitors of FtsZ were eliminated. Following this, two phytocompounds, Withaperuvin C and Trifolirhizin, were selected after the screening, demonstrating noteworthy binding potential with FtsZ’s GTP binding pocket, acting as potent GTP-competitive inhibitors of FtsZ. The study suggested that these compounds could be further investigated for developing a novel class of antibiotics after required studies.

## 1. Introduction

Filamenting temperature-sensitive mutant Z (FtsZ) is a highly conserved protein that plays a crucial role in bacterial cell division [1]. FtsZ is a member of the tubulin superfamily and shares structural and functional similarities with eukaryotic tubulin [2]. FtsZ is a key component of the bacterial cytokinetic machinery, which forms the Z-ring at the division site, a contractile ring responsible for cell division [3]. As such, FtsZ is essential for the growth and proliferation of bacteria and represents a target for antibacterial agents [4]. Bacterial cell division is a complex process that involves the formation of a septum to divide the cell into two daughter cells [5]. FtsZ is a key player in this process, forming a dynamic ring-like structure known as the Z-ring at the division site [6]. This Z-ring serves as a scaffold for recruiting other division proteins, leading to the synthesis of the septum and, eventually the separation of the daughter cells [7]. Targeting FtsZ in drug discovery holds great potential for developing new antibiotics and combating bacterial infections [8].

FtsZ comprises a single polypeptide chain that folds into a globular domain at the N-terminus, followed by a helical domain and a C-terminal tail [9]. The globular domain contains the GTPase activity of FtsZ, which is responsible for hydrolyzing GTP to GDP and phosphate regulating the polymerization and depolymerization of FtsZ filaments [10]. The helical domain is involved in the self-assembly of FtsZ into protofilaments, which further polymerize to form the Z-ring [11]. The C-terminal tail of FtsZ is involved in regulating protein-protein interactions and localization of FtsZ to the division site [12].

The interaction of FtsZ with various proteins is crucial for bacterial cell division, as it has been demonstrated to be an essential component of the process [13]. These include FtsA, a membrane-bound protein that interacts with FtsZ and is required for Z-ring assembly; ZipA, a transmembrane protein that anchors FtsZ to the inner membrane; and FtsE and FtsX, two GTP-binding proteins that are involved in the transport of cell wall precursors across the membrane during cell division [14, 15]. FtsZ also interacts with several other proteins involved in cell division, such as FtsK, a DNA translocase that is required for the segregation of chromosomes, and MinC, MinD, and MinE, proteins that are involved in regulating the placement of the division site [5, 16]. FtsZ is regulated by several factors, including the availability of GTP, which is required for polymerization, and the activity of proteins interacting with FtsZ [17]. For example, FtsA has been shown to stimulate the GTPase activity of FtsZ, which promotes the depolymerization of FtsZ filaments [18]. FtsZ is also regulated by post-translational modifications, such as phosphorylation and acetylation, which have been shown to affect FtsZ localization and function [19].

Due to its indispensability for bacterial proliferation and its prevalence across bacterial species, FtsZ has been recognized as a potential target for antibacterial agents [4, 20]. Several compounds that target FtsZ have been identified, including benzamide derivatives, stilbene derivatives, and pyrimidine derivatives [21, 22]. FtsZ polymerization can be inhibited by certain compounds that have demonstrated antibacterial activity against a wide range of bacterial species, including drug-resistant strains [23, 24]. However, despite this promising potential, the development of FtsZ inhibitors as antibacterials is still in the early stages, and additional research is required to optimize their effectiveness and selectivity [25–27].

Virtual screening is a fast and cost-effective drug discovery and design approach that has demonstrated high efficacy [28]. It is an important tool in drug discovery, helping identify potential therapeutic molecules and screen out any toxic or unwanted compounds that could impede drug development [29]. To achieve this, virtual screening typically involves a range of methods, including molecular docking, Pan-assay interference compounds (PAINS) filter, absorption, distribution, metabolism, excretion, and toxicity (ADMET), prediction of activity spectra for biologically active substances (PASS), and molecular dynamics (MD) simulations [30]. Virtual screening has proven to be an indispensable component of modern drug discovery and design efforts, playing a crucial role in identifying potential therapeutics for a wide range of diseases [31].

A multitier virtual screening approach was utilized in this study, wherein a library of ~12,000 plant-derived compounds sourced from the IMPPAT 2.0 library was employed [32], a well-curated database of medicinal plants and their chemical constituents with therapeutic properties. We focused on identifying potential inhibitors of FtsZ and used InstaDock [33] to screen for virtual binding partners based on their docking affinity. The top 10 hits were then evaluated based on docking scores, binding modes, and other values of drug likeliness. Further filters were applied using the SwissADME tool [34] to remove any PAINS patterns [35] and ADME studies [36]. Furthermore, the compounds were screened for toxicity using the pkCSM tool [37], followed by PASS [38] and all-atom MD simulations [28] for 200 ns.

## 2. Materials and methods

### 2.1 Computational tools and web sources

A multistep virtual screening process was carried out using multiple bioinformatics software and web resources [31]. We utilized the InstaDock tool for the initial virtual screening based on the molecular docking approach. To visualize the structures and generate 3D and 2D plots, we employed PyMOL [39] and Discovery Studio Visualizer 2021 Client [40]. Additionally, we used several online web servers to assist in our analysis, including SwissADME, pkCSM, and PASS server. IMPPAT 2.0 database was accessed to retrieve phytochemicals as a source of small molecules library.

### 2.2 Receptor and library preparation

To obtain the three-dimensional structure of FtsZ, we retrieved the PDB ID: 6LL6 from the RCSB Protein Data Bank [41]. The structure was then refined by removing co-crystallized water molecules using PyMOL software. We further performed energy minimization using the SwissPDBV [42] and validated the structure using the UCLA Saves 6.0 server (https://saves.mbi.ucla.edu/). To obtain the compounds library, we downloaded it from the IMMPAT 2.0 database, applying the Lipinski rule of five [43]. All the compounds in the library were saved in ’.pdbqt’ format for molecular docking screening in InstaDock software.

### 2.3 Molecular docking screening

Molecular docking is a computational technique that is commonly used to find the most appropriate compound that can bind to a specific protein target’s binding site [44]. This technique involves calculating the binding affinity and ligand efficiency values to ensure effective binding of the ligand to the target for further analysis [45]. Virtual screening is another method for drug discovery that enables the quick and efficient screening of a thousand compounds against a predefined target to identify potential hits and lead compounds based on their binding affinities and docked scores [46]. In this study, we utilized the InstaDock software for virtual screening, which blindly docked compounds from the library. We employed the 3-D structure file of the FtsZ protein to identify the most suitable binding partners in the library. The results folder was then analyzed by extracting the files to determine the best candidates for further investigation.

### 2.4 ADMET and PAINS prediction

In the field of drug discovery, it is important to identify compounds that exhibit desirable ADME properties, as well as low toxicity, to ensure their safety for therapeutic use [36]. In this study, we utilized two online tools, SwissADME and pkCSM, to screen the compounds based on their ADMET and PAINS patterns. SwissADME is a tool that predicts the ADME properties of small molecules, including their solubility, permeability, and oral bioavailability. pkCSM, on the other hand, predicts the pharmacokinetic properties of compounds, such as their toxicity and activity. To predict the carcinogenicity of the compounds, the CarcinoPred-EL webserver was utilized in this study [47]. By utilizing these tools, we were able to select compounds that met the correct PAINS and ADMET criteria for further analysis. This enabled us to focus on compounds that are likely to exhibit favorable druglike properties and could therefore be suitable candidates for further drug development.

### 2.5 PASS analysis

The PASS analysis is a method used to predict the biological properties of small molecules on the basis of their structures [38]. This prediction is based on the probabilities of the compound being active (Pa) or inactive (Pi) in a particular category. A greater Pa value indicates a greater likelihood of the compound being active in the corresponding category. In this study, we performed PASS analysis for the selected compounds and analyzed their associations with FtsZ.

### 2.6 Interaction analysis

After conducting the PASS analysis, we proceeded to assess how the chosen compounds would bind to the FtsZ binding pocket. We analyzed the docked out-files, which were generated from the results folder acquired from the InstaDock screening, to observe the interactions. For a visual depiction of the ribbon and electrostatic potential surface, we employed PyMOL, a widely used 3D molecular visualization software tool. 2D diagrams for each compound were produced using Discovery Studio Visualizer (version 2021 Client), a comprehensive drug discovery and design platform. We selected compounds based on their binding site in the FtsZ protein. The binding poses specifically interacting with the critical residues of FtsZ were selected for further analysis. Finally, all-atom MD simulations were performed to investigate the behavior and movement of atoms and molecules over time for the selected compounds with FtsZ.

### 2.7 MD simulation

MD simulation is a powerful computational physics approach that predicts the time-dependent behavior of atoms using Newton’s laws of motion [48]. The traditional method of MD simulation was computationally expensive and required supercomputers to perform heavy calculations. However, modern MD simulations utilize a force field, which is a mathematical function that estimates the potential energy of the system based on the atomic coordinates, to predict atomic interactions and estimate overall energy [28]. This method significantly reduces computational complexity and time, making it a valuable tool in various fields, including drug discovery and delivery [49, 50]. In this study, we utilized the GROMACS 2020 Beta simulation suite [51] with the GROMOS 54A7 force field [52] to prepare FtsZ and its complexes with the selected compounds for MD simulation. The PRODRG web server [53] was used to generate the force field parameters for the selected compounds, and the solvation was performed in a box of the SPC216 solvent model [54]. The steepest descent approach was implemented to minimize the energy and mitigate atom clashes across all systems. After performing energy minimization, we gradually increased the temperature of each system from 0 K to 300 K over 100 ps. Afterwards, NVT (constant number of particles, volume, and temperature) and NPT (constant number of particles, pressure, and temperature) equilibration were performed for 1000 ps to ensure system stability. A production run of 200 ns was then conducted for each system, during which various properties such as solubility, binding free energy, kinetic measures, and structural stability of the selected compounds with FtsZ were evaluated.

### 2.8 Principal component analysis and free energy landscape

Principal component analysis (PCA) is a technique widely used in various fields, such as biology, chemistry, and engineering, to reduce the complexity of multidimensional data [55]. PCA enables the analysis of conformational sampling and stability of a protein molecule before and after binding with compounds [56]. In this study, we utilized the GROMACS software’s *gmx covar*, *gmx anaeig* and *gmx sham* modules to perform PCA and free energy landscape (FEL) analysis of FtsZ before and after the compounds binding.

## 3. Results and discussion

### 3.1 Molecular docking screening

To identify potential hits for targeting FtsZ, we screened a total of 12,000 compounds from the IMPPAT parent database using the Lipinski rule of five filters. Next, we performed molecular docking screening using the InstaDock tool to obtain the binding affinities of each compound with FtsZ. The docked conformations and affinity scores were generated using a molecular docking approach, and compounds that exhibited considerable docking scores with the FtsZ binding site were filtered out. Based on their binding score, we selected the top 10 hits out of the 12,000 phytoconstituents. These hits displayed exciting binding potential with the affinity range of −9.5 kcal/mol to ≤ −9.0 kcal/mol with FtsZ (**Table 1**). All of the chosen hits demonstrated greater affinity than the reference inhibitor of FtsZ, Berberine [57] and PC190723 [58]. The study indicated that the selected hits showed appreciable docking scores, which can be analyzed further for therapeutic search.

**Table 1:** Top 10 hits screened out against FtsZ. LE, Ligand Efficiency (kcal/mol/non-H atom).

S. No.	Compound ID	Affinity kcal/mol	LE
1.	IMPHY010666	−9.5	0.29
2.	IMPHY011703	−9.5	0.27
3.	IMPHY011259	−9.4	0.30
4.	IMPHY014859	−9.4	0.30
5.	IMPHY010711	−9.2	0.27
6.	IMPHY010466	−9.2	0.26
7.	IMPHY010912	−9.1	0.28
8.	IMPHY012785	−9.1	0.30
9.	IMPHY012553	−9.0	0.32
10.	IMPHY011909	−9.0	0.26
11.	Berberine	−7.3	0.29
12.	PC190723	−7.0	0.30

### 3.2 ADMET properties

In order to find safe and effective small molecule therapeutics, it is important to consider multiple druglike factors such as ADMET [59]. SwissADME and pkCSM were utilized as computational tools to filter compounds based on PAINS, ADMET, and other relevant factors. All the selected compounds follow Lipinski rule of five with feasible physicochemical properties (**S1 Fig**). Out of the initial 10 hits generated from molecular docking screening, two compounds were chosen after undergoing ADMET analysis, as they showed appreciable ADMET properties without any PAINS and carcinogenic patterns (**S1 Table**). Two compounds, IMPHY010466 (Withaperuvin C) and IMPHY010912 (Trifolirhizin), were selected for further analysis as they exhibited no toxicity, as seen in **Table 2**. Both compounds showed better ADMET properties than the reference inhibitors, as they possess toxic and carcinogenic patterns. The intestinal absorption of the two screened phytoconstituents, Withaperuvin C and Trifolirhizin was predicted to be within the permissible range, indicating that these compounds have the potential to be absorbed effectively in the intestine. Additionally, the water solubility of these compounds was also found to be acceptable, which suggests that they can dissolve in water, a crucial property for drug molecules.

**Table 2:** ADMET parameters of the selected compounds.

S. No.	Compound ID	Absorption	Distribution	Metabolism	Excretion	Toxicity	Carcinogenicity
		GI Absorption	BBB permeation	CYP2C6 Inhibitor	OCT2 substrate	AMES/Hepatotoxicity	
1.	IMPHY010466	High	No	No	No	No	No
2.	IMPHY010912	High	No	No	No	No	No
3.	Berberine	Moderate	Yes	Yes	No	No	Yes
4.	PC190723	High	No	No	No	Yes	Yes

### 3.3 PASS analysis

The PASS analysis evaluated the biological properties of the identified hits, Withaperuvin C and Trifolirhizin. To ensure the effectiveness and therapeutic potential of small molecules, it is essential to assess their biological activities [60]. The PASS analysis provides Pa and Pi values for each molecule, where Pa signifies the probability of a molecule being active, and Pi represents the probability of being inactive for a particular activity. Both compounds were identified with desirable antibacterial properties through the PASS analysis. The Pa values of Withaperuvin C and Trifolirhizin ranged from 0,675 to 0,108, indicating their potential for specific biological activities **(Table 3).** This analysis enables the identification and selection of molecules based on their biological properties.

**Table 3:** Selected molecules with their biological properties.

S. No.	Compound ID	Phytochemical	Pa	Pi	Activity
1.	IMPHY010466	Withaperuvin C	0,412	0,044	Antiprotozoal
			0,365	0,058	Antifungal
			0,305	0,035	Antipruritic, non-allergic
			0,334	0,072	Dermatologic
			0,108	0,055	Antibiotic Anthracycline-like
2.	IMPHY010912	Trifolirhizin	0,675	0,008	Antiinfective
			0,632	0,015	Antifungal
			0,588	0,015	Antiprotozoal
			0,467	0,020	Antibacterial
			0,441	0,076	Antiinflammatory
3.	Berberine	N/A	0,274	0,070	Antibacterial
			0,231	0,016	Topoisomerase II inhibitor
			0,235	0,033	Antitussive, narcotic
			0,337	0,171	Membrane integrity agonist
			0,226	0,113	Angiogenesis inhibitor
4.	PC190723	N/A	0,605	0,015	Antiallergic
			0,599	0,016	Antiasthmatic
			0,221	0,149	Antiprotozoal (Coccidial)
			0,183	0,146	Angiogenesis inhibitor
			0,168	0,147	Antibacterial

### 3.4 Interaction analysis

**Fig 1:**
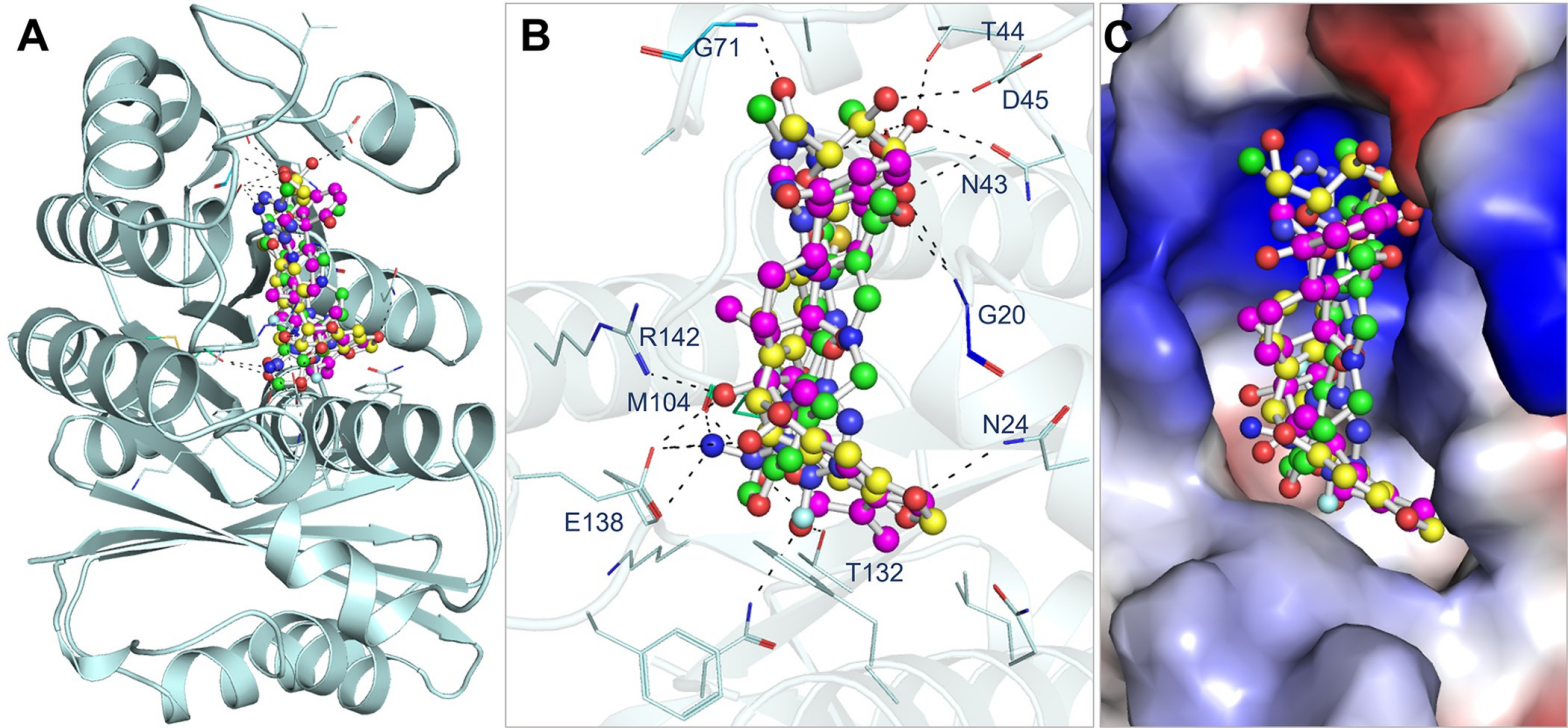
FtsZ interactions with Withaperuvin C, Trifolirhizin, Berberine, and PC190723. The binding pocket of FtsZ is shown in cyan, while the ligands are colored magenta, yellow, green, and blue, respectively. The magnified cartoon in B panel highlights the key interactions between the ligands and FtsZ residues. C panel shows the electrostatic view of FtsZ with Withaperuvin C (magenta), Trifolirhizin (yellow), Berberine (green), and PC190723 (blue). G20, N24, E138, and R142 are the GTP binding sites at FtsZ.

Withaperuvin C and Trifolirhizin compounds were evaluated for their interaction with FtsZ using PyMOL and Discovery Studio Visualizer (version 2021 Client). The docked conformation files of Withaperuvin C and Trifolirhizin were extracted from the out files and analyzed to identify their interactions with FtsZ. The GTP binding site is essential for the functional activity of FtsZ, and both compounds were observed to bind to this site. The 3D diagram revealed that Withaperuvin C and Trifolirhizin have complementarity with the GTP binding pocket cavity of FtsZ (**Fig 1**). **Fig 1A** shows the binding of FtsZ-Withaperuvin C and FtsZ-Trifolirhizin complexes, while **Fig 1B** explains the hydrogen bonding with the critical residues. The results showed that both compounds are superimposed to the binding of reference inhibitors that bind to the GTP binding site of FtsZ and exhibit interactions with important residues, such as Gly20, Asn24, Asn43, Thr44, Asp45, Gly71, Met104, Glu138, and Arg142. Additionally, **Fig 1C** shows the structural change in FtsZ after binding with Withaperuvin C and Trifolirhizin. These findings suggest that Withaperuvin C and Trifolirhizin have the potential to act as GTP-competitive inhibitors of FtsZ. This could pave the way for developing effective therapeutic agents for bacterial infections.

**Fig 2:**
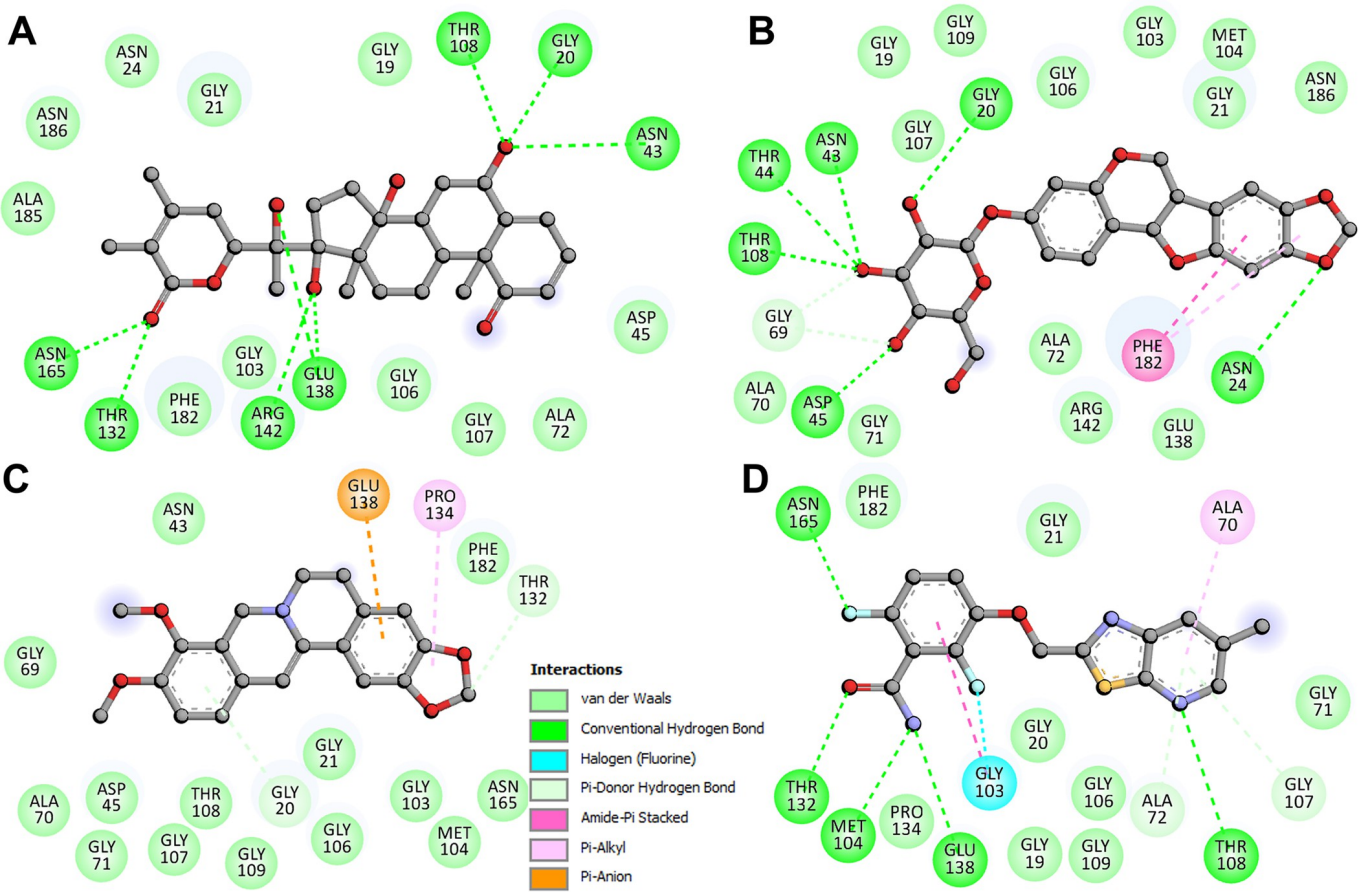
The 2D graphs demonstrating the FtsZ interactions with (**A**) Withaperuvin C and (**B**) Trifolirhizin, (C) Berberine, and (D) PC190723.

An extensive analysis of interactions was performed using Discovery Studio Visualizer 2021 Client to examine all possible interactions between Withaperuvin C and Trifolirhizin, as well as the reference inhibitors Berberine and PC190723, with FtsZ. 2D plots depicting all conceivable interactions for these compounds were generated (**Fig 2**). It was observed from the 2D diagram that both Withaperuvin C and Trifolirhizin bound to the GTP binding site of FtsZ with a close interaction with multiple bonds. The 2D plots showed that Withaperuvin C and Trifolirhizin interact with the GTP binding site residues and share the same interactions with the known FtsZ inhibitor, Berberine, and PC190723 (**Fig 2A–2D**). As a consequence, Withaperuvin C and Trifolirhizin may hamper FtsZ’s GTP availability, leading to the inhibition of its activity. These findings provide valuable insights into the molecular mechanism underlying the inhibitory effects of Withaperuvin C and Trifolirhizin on FtsZ and suggest their potential as drug candidates.

Overall, this analysis examined Withaperuvin C and Trifolirhizin’s interaction with FtsZ where both compounds were found to bind to FtsZ’s GTP binding site, displaying interactions with essential residues. We found that Withaperuvin C and Trifolirhizin establish hydrogen bonds, hydrophobic interactions, and other electrostatic interactions with specific residues that are essential for FtsZ’s function. The 3D and 2D diagrams revealed their close interactions, resembling known FtsZ inhibitors. For instance, residues such as Gly20, Asn24, Asn43, Thr44, Asp45, Gly71, Met104, Glu138, and Arg142 are known to be crucial for stabilizing the GTP-binding pocket of FtsZ, and our results indicate that both Withaperuvin C and Trifolirhizin establish key interactions with these residues. This suggests that Withaperuvin C and Trifolirhizin could function as GTP-competitive inhibitors, potentially leading to the development of effective therapeutics for bacterial infections. The extensive analysis of interactions with reference inhibitors further supported their potential as drug candidates, providing valuable insights into the molecular mechanisms of their inhibitory effects on FtsZ.

### 3.5 MD simulation

MD simulations were used to examine the thermodynamic properties and structural flexibility of molecular systems, FtsZ in its free state, FtsZ-Withaperuvin C, and FtsZ-Trifolirhizin. All-atom MD simulations were conducted for 200 ns to obtain precise estimates of the behavioral properties necessary for drug-target recognition. Several systematic parameters were used to assess the stability of FtsZ when interacting with Withaperuvin C and Trifolirhizin, which will be discussed in the following sections.

#### 3.5.1 Structural dynamics and compactness

We examined the structural and dynamic changes in FtsZ protein upon binding with Withaperuvin C and Trifolirhizin. To analyze these changes, we used the root mean square deviation (RMSD) and root mean square fluctuation (RMSF) methods, widely used in assessing structural changes in proteins after ligand binding. We first utilized the RMSD method to evaluate structural deviations and dynamics in FtsZ with Withaperuvin C and Trifolirhizin. The RMSD analysis showed that the average values of FtsZ, FtsZ-Withaperuvin C, and FtsZ-Trifolirhizin were 0.24 nm, 0.24 nm, and 0.26 nm, respectively. These values indicate that the protein structures remained stable without significant changes during the simulation time, which was 200 ns. Although we observed slight fluctuations in the RMSD plot of FtsZ-Trifolirhizin after 70 ns, these changes were insignificant. Furthermore, the probability distribution function (PDF) for the RMSD showed a small expansion in the RMSD of FtsZ with a similar probability over Trifolirhizin binding.

**Fig 3:**
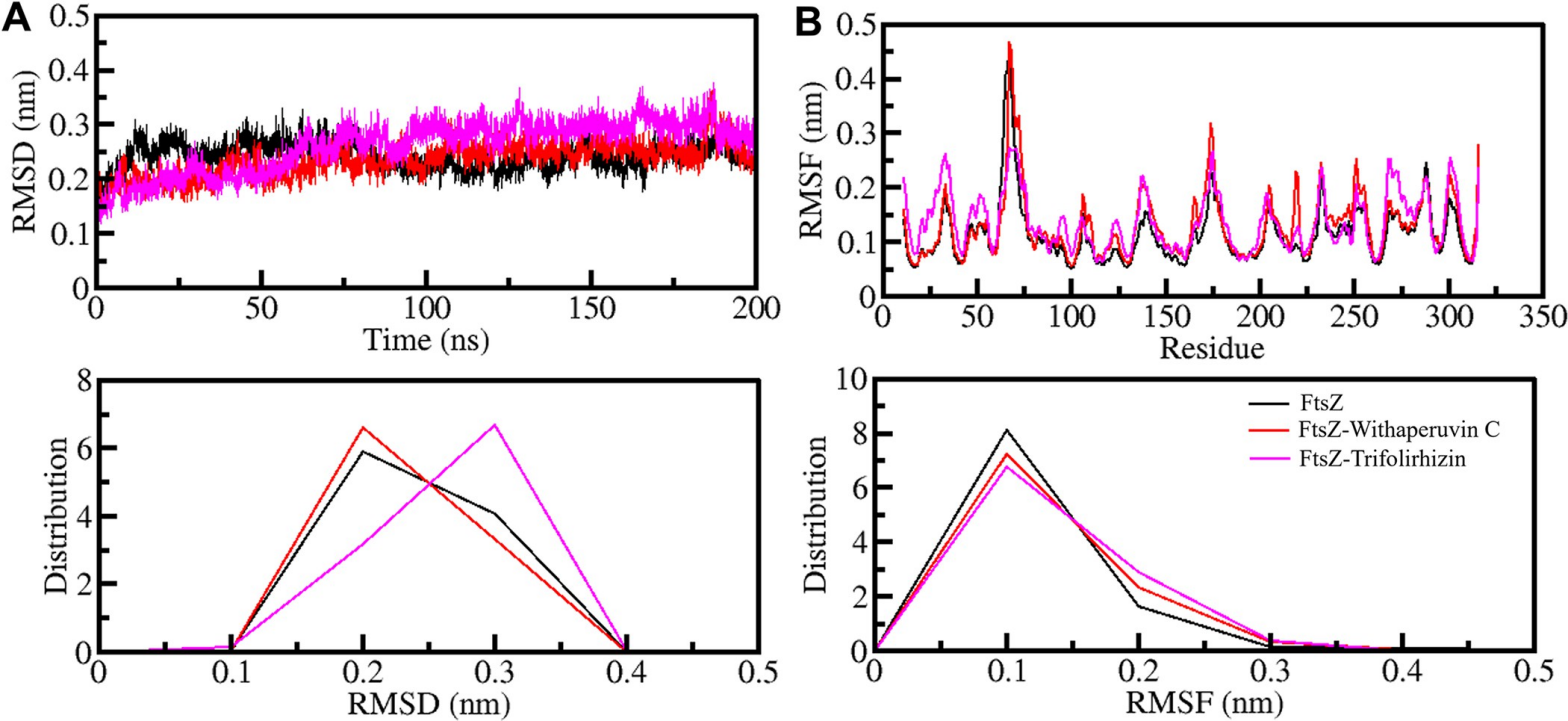
Structural dynamics of FtsZ with Withaperuvin C and Trifolirhizin. (**A)** RMSD and (**B)** RMSF plots of FtsZ with Withaperuvin C and Trifolirhizin. Lower panels exhibit the PDF values.

To understand the impact of the compounds binding on the FtsZ structure, we also used the RMSF method to measure residual fluctuations in FtsZ. The average RMSF values for FtsZ, FtsZ-Withaperuvin C, and FtsZ-Trifolirhizin were 0.12 nm, 0.13 nm, and 0.14 nm, respectively. The RMSF analysis revealed that FtsZ showed similar patterns of residual fluctuations before and after binding with the compounds, indicating the stability of the protein structure. The PDF also implied comparable residual fluctuations with a minor increase in FtsZ over the binding of Withaperuvin C and Trifolirhizin **(Fig 3B, lower panel)**. Overall, our findings suggest that the binding of Withaperuvin C and Trifolirhizin did not significantly alter the stability or dynamics of FtsZ. The RMSD and RMSF methods allowed us to assess the structural and dynamic changes in FtsZ protein at the atomic level.

**Fig 4:**
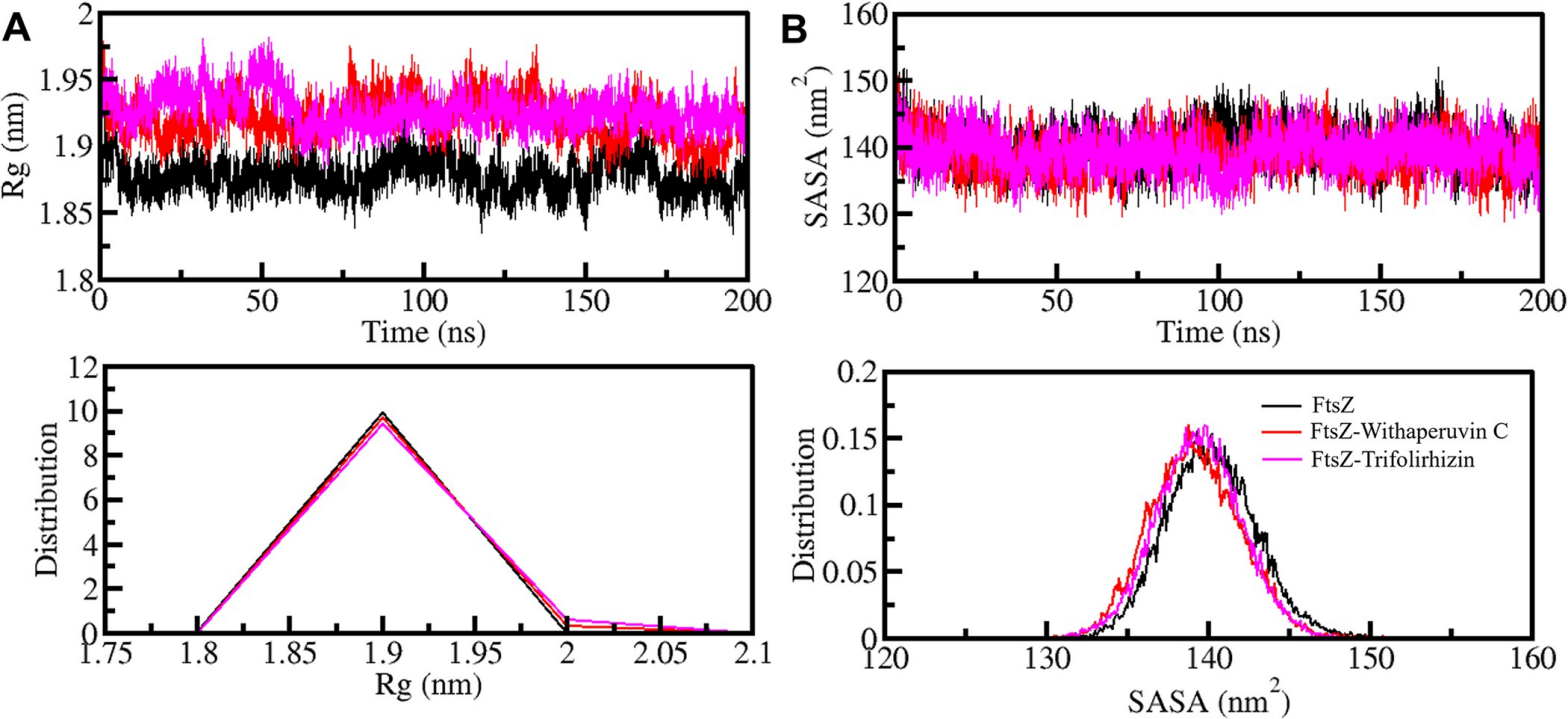
Compactness and folding parameters of FtsZ (**A)** *R*g plot and (**B)** SASA plot of FtsZ with Withaperuvin C and Trifolirhizin. Lower panels display the PDF values.

We used the radius of gyration (*R*g) to measure the compactness of the FtsZ structure and to assess any changes that may have occurred due to the binding of the ligands. We analyzed the FtsZ-Withaperuvin C and FtsZ-Trifolirhizin complexes using *R*g from the simulated trajectories (**Fig 4A**). The *R*g values for FtsZ, FtsZ-Withaperuvin C, and FtsZ-Trifolirhizin were 1.88 nm, 1.92 nm, and 1.93 nm, respectively. We found that the *R*g value of FtsZ-Withaperuvin C and FtsZ-Trifolirhizin was slightly higher than that of FtsZ-free. This observation suggests that the ligand binding leads to intermolecular occupancy, inducing a minor structural expansion of FtsZ by the compounds. The PDF of the *R*g values also showed that the FtsZ structure remained stable and folded in the ligand-bound states with Withaperuvin C and Trifolirhizin (**Fig 4A, lower panel**).

Solvent-accessible surface area (SASA) is a parameter that estimates the stability of biomolecules in different solvents in various conditions [61]. We used SASA values to study the stability of FtsZ and its docked complexes throughout the 200 ns simulation time. The average SASA values for FtsZ, FtsZ-Withaperuvin C, and FtsZ-Trifolirhizin were 140.16 nm^2^, 139.18 nm^2^, and 139.39 nm^2^, respectively. We found that the structures of FtsZ-Withaperuvin C and FtsZ-Trifolirhizin remained stable in the simulated trajectories. However, there was a slight decrease in SASA of FtsZ-Withaperuvin C and FtsZ-Trifolirhizin, with the overall protein folding (**Fig 4B**). The PDF graph also indicated a similar SASA distribution, further supporting the stability of these complexes **(Fig 4B lower panels)**.

#### 3.5.2 Dynamics of hydrogen bonds

**Fig 5:**
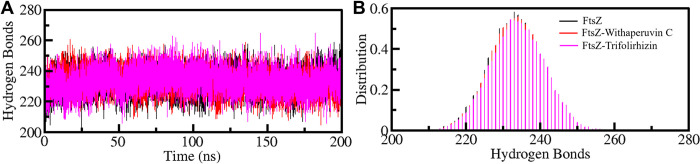
(**A**) Time progression of intramolecular hydrogen bonds in FtsZ. (**B**) The PDF of hydrogen bonding.

The formation of hydrogen bonds within the protein is crucial for maintaining the stability of the complex when it binds to ligands such as Withaperuvin C and Trifolirhizin. To investigate the temporal changes in intramolecular hydrogen bonding in FtsZ before and after complex formation, the simulated trajectories were analyzed (**Fig 5**). The analysis involved examining the hydrogen bond patterns within FtsZ both before and after it bound to Withaperuvin C and Trifolirhizin. The results were plotted on a graph, revealing no significant changes in the hydrogen bond patterns within FtsZ upon complex formation with Withaperuvin C and Trifolirhizin (**Fig 5A**). The average hydrogen bond values for FtsZ, FtsZ-Withaperuvin C, and FtsZ-Trifolirhizin were 233, 233, and 234, respectively. Specifically, the plot showed that the hydrogen bond formation within FtsZ remained constant even after binding with Withaperuvin C and Trifolirhizin (**Fig 5B**).

**Fig 6:**
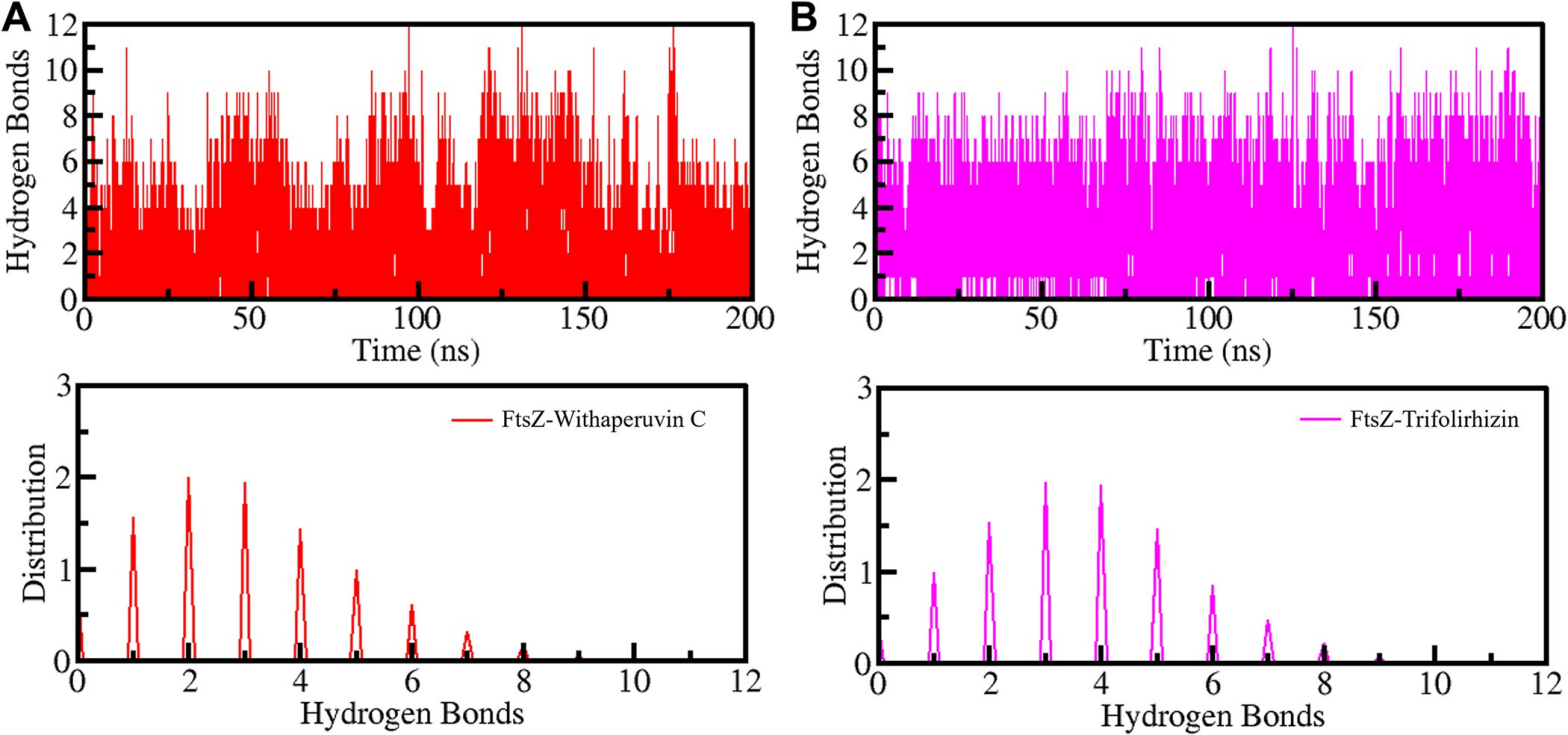
Time progression of intermolecular hydrogen bonds between FtsZ and (**A**) Withaperuvin C and (**B**) Trifolirhizin. Lower panels show the PDF values of the distribution.

The simulation trajectories were also scrutinized to evaluate the intermolecular hydrogen bonds established between the FtsZ protein and the ligands Withaperuvin C and Trifolirhizin (**Fig 6**). Our analysis revealed that the FtsZ-Withaperuvin C complex exhibited at least 3–5 stable intermolecular hydrogen bonds throughout the simulation time, indicating good stability (**Fig 6A**). In contrast, the FtsZ-Trifolirhizin complex showed 4–6 intermolecular hydrogen bonds, with some random fluctuations over the simulated time (**Fig 6B**). To further understand the stability of the hydrogen bonds in these complexes, we generated a PDF that showed the distribution of hydrogen bonds in both complexes (**Fig 6, lower panels**). The PDF analysis showed that the FtsZ-Trifolirhizin complex had a more stable pattern of hydrogen bonds than the FtsZ-Withaperuvin C complex. These results suggest that the FtsZ-Trifolirhizin complex is more likely to maintain stable interactions with the ligand than the FtsZ-Withaperuvin C complex, which showed more fluctuations. The analysis showed stable intermolecular hydrogen bonds between FtsZ and Withaperuvin C and Trifolirhizin, despite the ligands not moving significantly from their initial positions. The observations offer significant insights into the molecular interactions between the FtsZ protein and these ligands, potentially facilitating the development of innovative therapeutic agents.

### 3.6 PCA and FEL analysis

**Fig 7:**
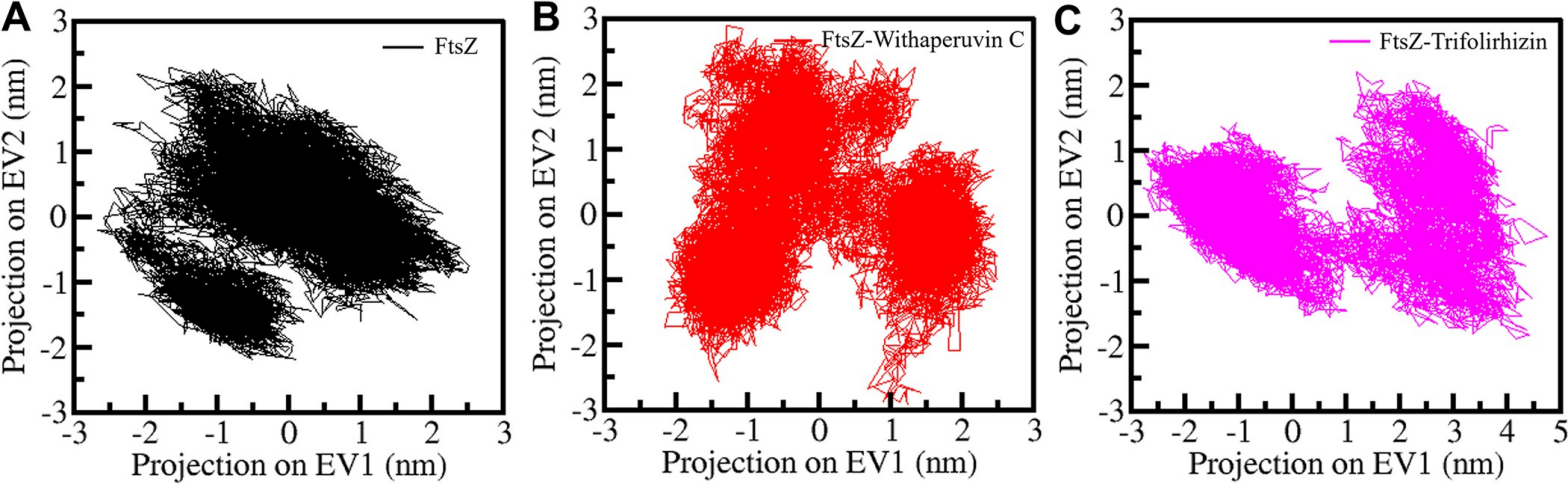
Conformational projections of (**A**) FtsZ, (**B**) FtsZ-Withaperuvin C and (**C**) FtsZ-Trifolirhizin.

To investigate the collective motions of FtsZ protein, FtsZ-Withaperuvin C, and FtsZ-Trifolirhizin complexes during the simulation, we performed PCA in this study. We used the essential dynamics approach to analyze the simulated trajectories, and the results showed that the FtsZ protein in its free state was covered by the FtsZ-Withaperuvin C and FtsZ-Trifolirhizin projections on two different eigenvectors (EVs) by its C_α_ atoms (**Fig 7**). The projections revealed that the conformations of FtsZ-Trifolirhizin were highly positively correlated on EV1 compared to FtsZ and FtsZ-Withaperuvin C (**Fig 7C**). As a whole, both complexes exhibited stable projections without any significant changes. Our analysis of the collective motions of FtsZ, FtsZ-Withaperuvin C, and FtsZ-Trifolirhizin complexes using PCA provides insights into the essential dynamics of these complexes. The PCA findings suggest that the collective motions of FtsZ protein and its complexes with Withaperuvin C and Trifolirhizin were stable throughout the simulation time.

**Fig 8:**
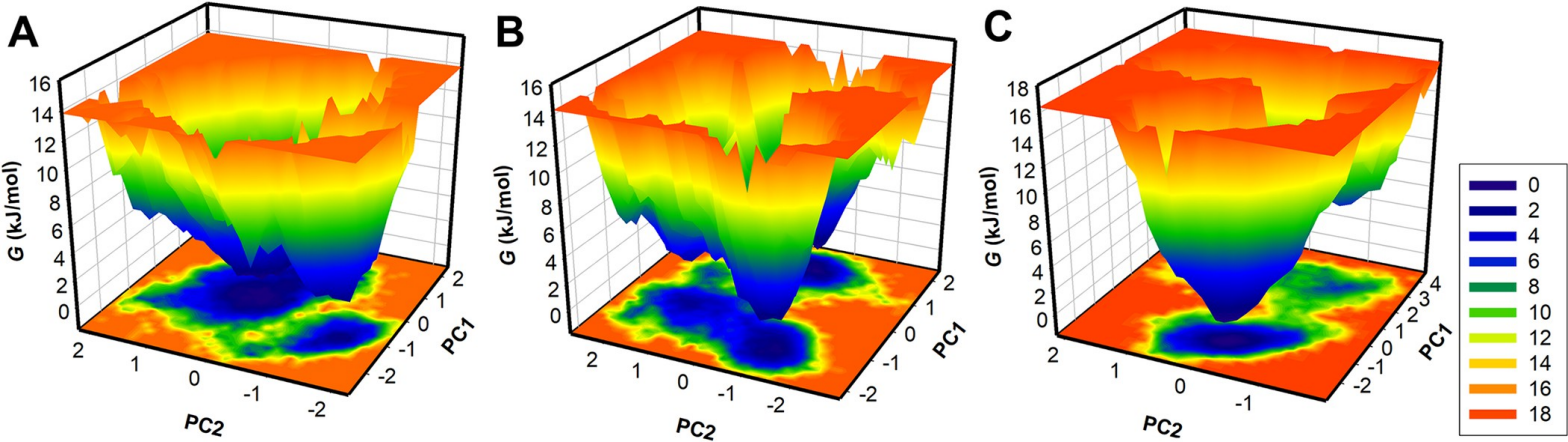
Free energy landscapes of (**A)** FtsZ (**B)** FtsZ-Withaperuvin C, and (**C)** FtsZ-Trifolirhizin.

The FEL is another commonly used method to examine how ligand binding affects protein folding and folding mechanism [56]. It provides valuable insights into the conformational changes and stability of the protein upon ligand binding. By generating FELs, we can observe the protein’s global minima and folding mechanism during simulation, helping us understand how ligand binding affects the protein’s structure. To assess the impact of Withaperuvin C and Trifolirhizin binding on FtsZ, we generated FELs to observe the protein’s global minima and folding mechanism from the simulated trajectory. **Fig 8** displays the contour FELs of FtsZ, FtsZ-Withaperuvin C, and FtsZ-Trifolirhizin, where deeper blue corresponds to the protein conformation being closer to the native state. FtsZ in the unbound state had a single global minimum and was limited to a large basin (**Fig 8A**). However, when bound to Withaperuvin C and Trifolirhizin, FtsZ showed slightly altered size and position of the phases and expanded to 2–3 global minima, as observed in the FELs.

The binding of Withaperuvin C and Trifolirhizin resulted in FtsZ acquiring different states with multiple basins. Still, it did not cause the unfolding of FtsZ during the simulation, indicating that FtsZ remained stable after ligand binding (**Fig 8B and 8C**). These findings suggest that the binding of Withaperuvin C and Trifolirhizin did not significantly affect the folding stability of FtsZ. In summary, the FEL approach used in this study provided valuable insights into the global minima and folding mechanism of FtsZ, FtsZ-Withaperuvin C, and FtsZ-Trifolirhizin complexes and suggested that the binding of these ligands did not induce any significant conformational changes in FtsZ. Overall, FEL analysis aids in understanding the structural changes induced by ligand binding and helps us assess the impact of ligands on protein stability and folding mechanisms.

## 4. Conclusion

The emergence of bacterial resistance has created an urgent need to develop innovative antibacterial drugs. FtsZ has garnered attention as a potential target for developing antibiotics that can impede bacterial cell division and arrest bacterial growth. In this study, we conducted a virtual screening of phytochemicals from the IMPPAT 2.0 library and identified two compounds, Withaperuvin C and Trifolirhizin, as potential inhibitors of FtsZ. The virtual screening process involved multiple steps, including Lipinski rule of five, molecular docking, PAINS filter, ADMET, PASS analysis, and all-atom MD simulations followed by PCA and FEL analyses, to eliminate compounds that could have adverse effects and to identify compounds with significant binding to FtsZ’s GTP binding pocket. The selected compounds demonstrated potent GTP-competitive inhibition of FtsZ. The results suggest that Withaperuvin C and Trifolirhizin have the potential to serve as lead compounds for further optimization and development into potential therapeutics for bacterial infections and other diseases involving FtsZ.

In addition, the study provides insights into the interactions of Withaperuvin C and Trifolirhizin with FtsZ and their potential mechanisms of action. The MD simulation showed that both Withaperuvin C and Trifolirhizin formed stable complexes with FtsZ, with Withaperuvin C exhibiting higher stability and binding affinity than Trifolirhizin. The binding of these compounds to FtsZ’s GTP binding pocket could interfere with the formation of the Z-ring and, therefore, inhibit bacterial cell division. Overall, this study demonstrates the potential of virtual screening approaches to identify new inhibitors of FtsZ and highlights the importance of further exploring natural compounds as a source of lead compounds for drug development. Further optimization and characterization of Withaperuvin C and Trifolirhizin could lead to the development of novel antibiotics and therapeutic agents for bacterial infections and other diseases associated with FtsZ. In summary, while the virtual screening and molecular dynamics simulations provide valuable insights and potential lead compounds, the study’s findings need further validation through in-vitro and in-vivo experimentation. Additionally, the challenges of translating the results to the clinic, including safety, bioavailability, and efficacy, must be thoroughly addressed before considering these compounds as potential therapeutic agents for bacterial infections and other diseases involving FtsZ.

## Supporting information

S1 Fig Radar plot showing the physicochemical properties of the selected compounds against FtsZ calculated through SwissADME server (http://www.swissadme.ch/index.php). (PPTX)

S1 Table Physicochemical and ADMET properties of the selected compounds against FtsZ. (XLSX)
