# Supplementary material for: Targeting Filamenting temperature-sensitive mutant Z (FtsZ) with bioactive phytoconstituents: An emerging strategy for antibacterial therapy
Source: PLoS One. 2023 Aug 30;18(8):e0290852. doi: 10.1371/journal.pone.0290852 (PMC10468062; doi:10.1371/journal.pone.0290852)
Supplement: S1 Fig — (PPTX) [file pone.0290852.s001.pptx]

## Slide 1
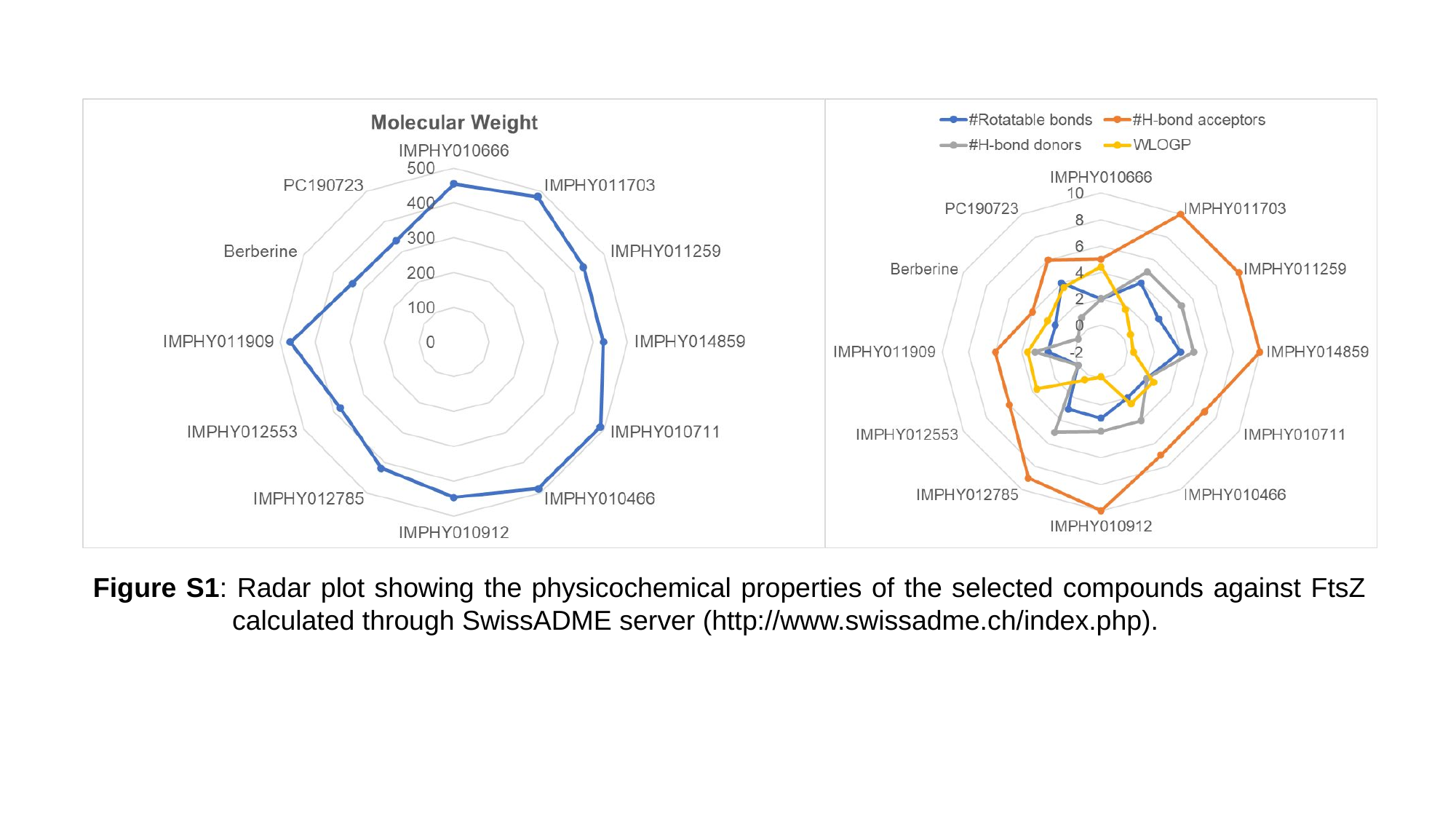

Figure S1: Radar plot showing the physicochemical properties of the selected compounds against FtsZ calculated through SwissADME server (http://www.swissadme.ch/index.php).
